# A Safety Evaluation of N-Acetylglucosamine Produced by *Bacillus subtilis* BNZR 2-7: A Comprehensive In Vitro and In Vivo Genotoxicity Assessment

**DOI:** 10.3390/biom15101368

**Published:** 2025-09-26

**Authors:** Liangliang Zhang, Jiahui Hao, Xuefang Liu, Kualian Yang, Lina Bai, Qingping Hu, Saisai Feng

**Affiliations:** 1 School of Food Science, Shanxi Normal University, Taiyuan 030031, China; 703581@sxnu.edu.cn (L.Z.); 223112001@sxnu.edu.cn (J.H.); liuxuefang@sxnu.edu.cn (X.L.); yangkualian@sxnu.edu.cn (K.Y.); 225420004@sxnu.edu.cn (L.B.); 2 College of Life Science, Shanxi Normal University, Taiyuan 030031, China; 315042@snux.edu.cn

**Keywords:** N-acetylglucosamine, *Bacillus subtilis*, genotoxicity assessment, mutagenic activity, micronucleated cells, chromosomal aberrations

## Abstract

N-acetylglucosamine (GlcNAc) is an important functional monosaccharide that serves as a key component in macromolecules such as cell walls and chitin. It has a wide range of applications in medicine, health supplements, and the chemical industry, leading to a growing market demand. This study evaluated the potential genotoxicity of GlcNAc produced by *Bacillus subtilis* BNZR 2-7 through a comprehensive assessment in vitro and in vivo assays. GlcNAc was non-mutagenic in the Ames test using *Salmonella typhimurium* and *Escherichia coli*. Meanwhile, GlcNAc was non-genotoxic in the in vitro micronucleus assay using Chinese hamster ovary cells. In the in vivo assays, GlcNAc was non-genotoxic in the in vivo mammalian erythrocyte micronucleus test and spermatocyte chromosome aberration test in mice. These studies provide additional evidence that GlcNAc produced by *Bacillus subtilis* BNZR 2-7 is not genotoxic at the doses tested, supporting its safety for use in foods.

## 1. Introduction

N-acetylglucosamine (GlcNAc) is a functional monosaccharide. It is formed when the second hydroxyl group of a glucose molecule is replaced by an acetyl amino group [1]. It is a fundamental building block of various functional polysaccharides and also participates in numerous physiological processes within the body [2]. As an important component of cartilage and synovial fluid, GlcNAc can promote joint repair and alleviate joint pain, showing significant efficacy in treating osteoarthritis and joint injuries [3]. Additionally, GlcNAc does not impact blood glucose levels, highlighting its potential as a promising low-calorie and health-conscious sugar substitute for individuals with diabetes [4]. GlcNAc also promotes the growth and proliferation of beneficial gut bacteria, thereby helping to alleviate gastrointestinal inflammatory diseases [4,5]. Moreover, studies have shown that GlcNAc promotes mucopolysaccharide synthesis in the human body and inhibits liver cancer cells’ growth, metastasis, and spread [6,7]. Therefore, it has also shown potential in cancer control [8,9,10]. As a building block of hyaluronic acid, GlcNAc can also promote glycosaminoglycan synthesis in the human body, increase hyaluronic acid levels in the skin, and improve issues like dryness and roughness caused by aging [11]. As research on GlcNAc deepens, more functions are being discovered. The broad application prospects and growing market demand highlight the potential of GlcNAc [4,12].

Currently, there are three main production methods for GlcNAc, including the chemical synthesis method [4], enzymatic synthesis method [4], and microbial fermentation method [13]. However, salt ions originating from glucosamine hydrochloride or glucosamine sulfate produced by the chemical method can cause stomach discomfort as well as liver and kidney damage [4]. Additionally, GlcNAc derived from crustacean shells and other chitin sources may trigger allergic reactions and produce large amounts of wastewater during production, thus harming the environment [14]. Therefore, the microbial fermentation method has gradually attracted more attention from researchers. This method is not limited by raw materials, offers high production efficiency, reduces pollution, and does not cause allergic reactions. The synthesis of GlcNAc by microbial fermentation typically uses glucose as raw materials. Currently, *Escherichia coli* is the primary microorganism used for GlcNAc production; however, its endotoxins, acetic acid byproducts, and vulnerability to phage contamination restrict its practical use [15,16]. Therefore, it is necessary to investigate safer and more efficient microorganisms for the actual production of GlcNAc.

Unlike *Escherichia coli*, *Bacillus subtilis* has long been used as a production vector, which produces biochemical substances with significant medicinal value as well as industrial components that meet GRAS standards [17]. In recent years, it has become an important raw material for producing GlcNAc [18]. Recently, GlcNAc has been approved and marketed in multiple countries and regions worldwide, including China, Canada, and Korea, and has been widely used in fields such as food, medicine, and cosmetics [19]. However, the safety of N-acetylglucosamine made by *Bacillus subtilis* remains unproven. In addition, although GlcNAc is regarded as non-toxic to humans judging from the results of some toxicity studies, systematic safety evaluation for genotoxicity has been lacking.

In this study, we evaluated the genotoxic safety of GlcNAc, which was produced by microbial fermentation using *Bacillus subtilis* BNZR 2-7 with a purity of ≥98%. *Bacillus subtilis* BNZR 2-7 was constructed by using *Bacillus subtilis* 168 as the parental strain for genetic modification. A series of assays was conducted to assess the potential genotoxicity of the produced GlcNAc, including a bacterial reverse mutation assay (Ames test), in vitro micronucleus assay in Chinese hamster ovary cells, in vivo mammalian erythrocyte micronucleus test, and spermatocyte chromosome aberration test in mice. The results support the safety of high-purity GlcNAc (≥98%) for use in food applications.

## 2. Materials and Methods

### 2.1. Animals

Institute of Cancer Research (ICR) mice of specific pathogen-free (SPF) grade were obtained from Hangzhou Medical College (Hangzhou, China). The test animal feed was obtained from Hangzhou Medical College (Hangzhou, China). The testing environmental conditions were as follows: temperature 20–25 °C and relative humidity 40–70%.

### 2.2. N-Acetylglucosamine

The test sample, N-acetylglucosamine (a white or off-white crystalline powder with a purity ≥ 98%), was produced by *Bacillus subtilis* BNZR 2-7 through microbial fermentation and was provided by Shandong Runhan Biotechnology Co., Ltd. (Zibo, Shandong, China).

### 2.3. Bacterial Reverse Mutation Assay (Ames Test)

*Salmonella typhimurium* histidine auxotrophs TA98, TA100, TA1535, and TA1537, as well as S9 mix (metabolic activation system; rat liver post-mitochondrial supernatant induced with phenobarbital and 5,6-benzoflavone), were obtained from Molecular Toxicology, Inc. (Moltox, Boone, NC, USA). *Escherichia coli* tryptophan auxotroph WP2 uvrA originated from the National Collections of Industrial and Marine Bacteria, Aberdeen, Scotland, UK.

The test was carried out according to OECD test guideline 471 and Guidance on genotoxicity testing and data interpretation for pharmaceuticals intended for human use (ICH S2 (R1)) [20]. The tester strains used were the *Salmonella typhimurium* histidine auxotrophs TA98, TA100, TA1535, and TA1537 and the *Escherichia coli* tryptophan auxotroph WP2 uvrA. A test article is considered positive if it induces a biologically relevant, concentration-related increase in the mean number of revertants per plate in at least one tester strain, observed at a minimum of two increasing concentrations of the test article. Specifically, a ≥3.0-fold increase over the concurrent solvent/negative control is required for strains TA1535 and TA1537, whereas a ≥2.0-fold increase is required for strains TA98, TA100, and WP2 uvrA.

Overnight cultures were prepared to use them for the mutagenicity assay, and they were inoculated from the frozen working stock. A total of 20 µL of the frozen stock was added for every 5 mL of Oxoid nutrient broth No. 2. For tester strains TA98 and TA100, 62.5 μL of 0.8% ampicillin solution was added into 20 mL of Oxoid nutrient broth No. 2. The strain and nutrient mixtures were then incubated with shaking set at 130 rpm and at 37 °C until an optical density of 0.6 to 1.6 (OD_650_ value) was achieved. Cultures were removed from incubation when the target density was reached and were held at 2 °C to 8 °C until used in the assay.

The dose levels of GlcNAc at 50, 100, 250, 500, 1000, 2500, and 5000 μg/plate were selected for the definitive mutagenicity assay based on the results of the dose range-finding study, which showed no observable cytotoxicity toward the tester strains and no precipitation up to 5000 μg/plate. The assay was performed in both the presence and absence of S9 mix, along with concurrent solvent/negative controls (sterile water for injection, which was used as the solvent for GlcNAc) and positive controls (the positive controls for each tester strain are presented in Table 1). Tester strains were exposed to the test article using the plate incorporation method. Briefly, in this method, the test article, tester strain, and S9 mix (when applicable) were combined and added to molten top agar, which was then overlaid onto a minimal glucose agar plate. After incubation, revertant colonies were counted. All dose levels of GlcNAc, as well as solvent/negative and positive controls, were plated in triplicate.

### 2.4. In Vitro Micronucleus Assay in Chinese Hamster Ovary Cells

Chinese hamster ovary cell line (CHO-WBL) cells were originally obtained from Sigma-Aldrich (St. Louis, MO, USA). The cell stocks were stored in liquid nitrogen. Every batch of the cell stocks was checked for the stability of the modal chromosome number and was tested and determined to be free from mycoplasma contamination. Cells were not used after the 32nd passage. The Lot number of cells used in this study was 20230310-WuXi GIVT.

The test was carried out according to OECD test guideline 487 and Guidance on genotoxicity testing and data interpretation for pharmaceuticals intended for human use (ICH S2 (R1)) [20]. GlcNAc was evaluated for the clastogenic/aneugenic potential in this test using Chinese hamster ovary (CHO-WBL) cells in the absence of activation systems for 3 and 24 h and in the presence of S9 mix for 3 h. Based on the results of the concentration range-finding assay, and in the absence of limiting cytotoxicity or solubility, 10 mM was selected as the highest concentration in accordance with U.S. FDA Redbook 2000, Chapter IV.C.1.b [21]. Accordingly, the following concentrations were evaluated in the definitive micronucleus assay across all three treatment series: 275, 550, 1100, and 2212.1 μg/mL (Table 2). Sterile water, used as the solvent for GlcNAc, served as the negative control. In parallel, cells treated with 2.5 μg/mL cyclophosphamide monohydrate, 0.5 μg/mL mitomycin C, and 0.2 μg/mL colchicine were included as positive controls.

Target cells were prepared, and exponentially growing CHO-WBL cells were seeded in complete medium for each treatment condition at approximately 0.4 × 10^6^ viable cells/25 cm^2^ flask with 5 mL medium. Two extra cultures were set up for the determination of baseline cell counts at the time of treatment. The flasks were incubated in a humidified incubator of 5% (4% to 8%) CO_2_ in air at 37 °C (±2 °C) for 20 to 24 h.

Cultures of CHO-WBL cells were exposed in duplicates to 4 concentrations of the test article and to positive and negative controls. In the S9-activated treatment series, treatment was for 3 h; in the non-activated treatment series, treatment was for 3 h and for 24 h. For the S9-activated 3 h treatment series and the non-activated 3 h treatment series, the cells were treated for 3 h (±15 min). At the end of the treatment period, cultures were examined by eye/microscope for precipitation. For the removal of the test article, the treatment medium was aspirated. The cells were washed twice with Hank’s buffered salt solution (HBSS), refed with 5 mL complete medium, and cultured for 21 h (±30 min) until harvest. For the non-activated 24 h treatment series, the cells were treated for 24 h (±30 min).

Cells were harvested by trypsinization for 24 h (±30 min) after the initiation of treatment, and an aliquot was taken for counting using an automated cell analysis system. The remainder of the cells were pre-fixed and then washed with two consecutive changes of fixative (anhydrous methanol–glacial acetic acid, 3:1 *v*/*v*). To prepare slides, the cells were collected by centrifugation. An aliquot of fixed cells was applied drop-wise onto a microscope slide. Two slides were prepared from each culture (one was for scoring, and the other was for backup). Each slide was identified. The slides were stained with acridine orange and air-dried. Under the microscope, a total of 2000 cells from each concentration were examined.

### 2.5. In Vivo Mammalian Erythrocyte Micronucleus and Spermatocyte Chromosome Aberration Tests

The in vivo mammalian erythrocyte micronucleus and spermatocyte chromosome aberration tests were carried out according to Chinese national standards GB 15193.5-2014 and GB 15193.8-2014 [22,23]. The mice were fed under strict specific pathogen-free (SPF) conditions at the Zhejiang Academy of Medical Sciences. All experimental procedures involving the mice were ethically reviewed and approved by the Institutional Animal Care and Use Committee, Zhejiang Academy of Medical Sciences (Approval number: ZJCLA-IACUC-20100008).

For the in vivo mammalian erythrocyte micronucleus test, SPF-grade mice (25–30 g) comprising 25 males and 25 females were obtained from the Institute of Cancer Research (ICR). The animals were randomly divided into five groups, with 10 mice per group and an equal distribution of males and females. The test included three dose groups, 2.5, 5.0, and 10.0 g/kg BW, which was based on the result of the acute oral toxicity test (LD_50_ > 20 g/kg BW for mice; half of this value was taken as the high-dose group in accordance with GB 15193.5-2014), along with the negative control (distilled water, which was used as the solvent for GlcNAc) and the positive control group (cyclophosphamide 40 mg/kg BW). The test sample was given orally by gavage at a dose of 20 mL/kg BW two times with an interval of 24 h. Sternal bone marrow samples were collected 6 h after the final dose for slide preparation. At least 200 red blood cells per mouse were examined to calculate the proportion of polychromatic erythrocytes (PCEs) in the total red blood cells (RBCs), and 2000 polychromatic erythrocytes per mouse were examined to calculate micronucleus incidence.

For the spermatocyte chromosome aberration test, 25 specific pathogen-free (SPF)-grade male mice (25–35 g) were obtained from the Institute of Cancer Research (ICR) and randomly divided into 5 groups, with 5 mice per group. The test included three dose groups, 2.5, 5.0, and 10.0 g/kg BW, which was based on the result of the acute oral toxicity test (LD_50_ > 20 g/kg BW for mice; half of this value was taken as the high-dose group in accordance with GB 15193.8-2014), along with the negative control (distilled water, which was used as the solvent for GlcNAc) and the positive control group (cyclophosphamide 40 mg/kg BW). The test animals in each dose group were given the test sample orally by gavage at a dose of 20 mL/kg BW for 5 consecutive days, once per day. The test animals were sacrificed on the 12th day after the first administration of the test sample. Four hours before the test animals were sacrificed, colchicine at a dose of 6 mg/kg BW was intraperitoneally injected. The test animals were sacrificed by cervical dislocation. The testicles on both sides of the test animals were taken for hypotonic treatment, the separation of seminiferous tubules, and other operations and then fixed twice with fixative, centrifuged conventionally, and the supernatant was removed, and the slides were dried at low temperature and subsequently stained with Giemsa for slide preparation. Mitotic phase cells were observed under the microscope. Each mouse was counted to 100 metaphase mitotic phase cells, and 500 metaphase mitotic phases were observed in each dose group.

### 2.6. Statistics

In the bacterial reverse mutation assay, the mean number of revertant colonies, standard deviations, and mutation rates (defined as the ratio of the mean number of revertant colonies in the treatment or positive control groups to that in the solvent control group) were calculated with R software (4.2.1). In the in vitro micronucleus assay using Chinese hamster ovary cells, Fisher’s exact test was applied to detect statistically significant differences in the frequency of micronucleated cells between the test article-treated groups or the positive control group and the negative control group. The Cochran–Armitage test was used to measure concentration responsiveness. In the in vivo mammalian erythrocyte micronucleus test, the mean and standard deviation of the micronucleus incidence in each group were analyzed separately by sex. Comparisons between each dose group and the negative control group were performed assuming a Poisson distribution. In the spermatocyte chromosome aberration test in mice, the number and frequency of different types of chromosome structural aberrations in each group were recorded. The rate of aberration cells, autosomal monosomy, and sex chromosome monosomy were compared by a two-tailed Welch’s *t*-test. A *p*-value of ≤0.05 was considered statistically significant.

## 3. Results

### 3.1. GlcNAc Did Not Exhibit Mutagenic Activity in Bacterial Species

The results of the bacterial reverse mutation assay are presented in Table 3. No precipitate was observed (by visual inspection after the incubation period) at any dose level of GlcNAc, either with or without S9 mix, in any tester strain. As shown in Table 3, no cytotoxicity was observed at any concentration of GlcNAc, with or without S9 mix, in any tester strain. Across all tested concentrations, GlcNAc did not cause a biologically relevant or concentration-dependent increase in revertant colonies. Under both metabolic activation (+S9) and non-activation (−S9) conditions, revertant counts for all strains remained comparable to the concurrent negative controls, with the exception of TA100 (−S9), which showed a minor but statistically significant decrease (*p* < 0.05). Importantly, GlcNAc did not exceed the threshold criteria of a 2-fold increase (TA98, TA100, WP2 uvrA) or 3-fold increase (TA1535, TA1537) in revertant numbers relative to the negative control, and no dose–response was observed. In contrast, all positive controls produced the expected increases (>3-fold, *p* < 0.0001). These results demonstrate that GlcNAc did not induce base-pair substitutions or frameshift mutations in any of the tester strains and therefore showed no mutagenic activity under the conditions of this study.

### 3.2. GlcNAc Was Negative for the Induction of Micronucleated Cells in CHO-WBL Cells

The summarized results of the in vitro micronucleus assay in Chinese hamster ovary cells are presented in Table 4. No precipitate was observed in treatment medium at any concentration during treatment. As no limiting precipitation or cytotoxicity was observed, the lowest concentration was not needed for allocation to microscopic examination for micronuclei based on the recommendations of OECD 487 [24]. Therefore, the maximum concentration (2212.1 µg/mL) and the two lower concentrations of 1100 and 550 µg/mL were used in accordance with the recommendations of OECD 487 and U.S. FDA Redbook 2000, Chapter IV.C.1.b. [21].

**Figure 1:**
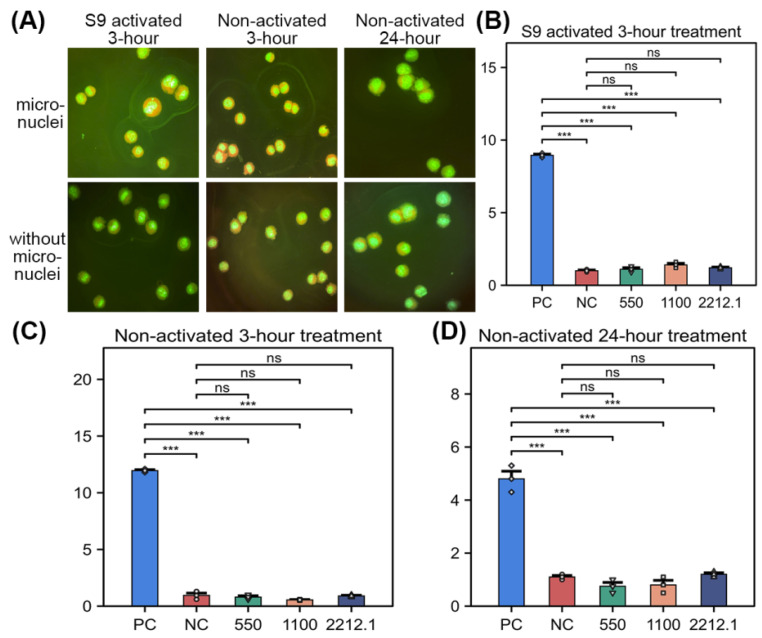
In vitro micronucleus assay in Chinese hamster ovary cells exposed to GlcNAc. (**A**) The micronucleus assay in Chinese hamster ovary cells with different experimental groups. (**B**–**D**) The ratio of micronucleated cells with S9-activated 3 h treatment (**B**), non-activated 3 h treatment (**C**), and non-activated 24 h treatment (**D**). A two-tailed Welch’s *t*-test was used to compare means between two independent groups, *** *p* < 0.001. ns, not significant (*p* > 0.05).

For the S9-activated 3 h treatment series, the mean frequency of cells with micronuclei in the concurrent negative control treatment group was 1.00%, and the increase in the average frequency of cells with micronuclei relative to the concurrent negative control group in the cyclophosphamide monohydrate (positive control) treatment group (8.95%) was statistically significant (*p* ≤ 0.05, Figure 1A,B). No statistically significant increase in the percentage of cells with micronuclei in the test article-treated group was observed at any concentration relative to the concurrent negative control group (*p* > 0.05, Figure 1A,B); the frequency of micronucleated cells was within the negative control data range reported in the literature (0.0–2.20%); and a concentration-related response was not observed. Based on these results, GlcNAc was concluded to be clearly negative for the induction of micronuclei in the test system of 3 h exposure in the presence of S9 metabolic activation.

For the non-activated 3 h treatment series, the mean frequency of cells with micronuclei in the concurrent negative control treatment group was 0.95%, and the increase in the average frequency of cells with micronuclei relative to the concurrent negative control group in the mitomycin C (positive control) treatment group (11.95%) was statistically significant (*p* ≤ 0.05, Figure 1A,C). No statistically significant increase in the percentage of cells with micronuclei in the test article-treated group was observed at any concentration relative to the concurrent negative control group (*p* > 0.05, Figure 1A,C); the frequency of micronucleated cells was within the negative control data range reported in the literature (0.0–2.20%); and a concentration-related response was not observed. Based on these results, GlcNAc was concluded to be clearly negative for the induction of micronuclei in the test system of 3 h exposure in the absence of S9 metabolic activation.

For the non-activated 24 h treatment series, the mean frequency of cells with micronuclei in the concurrent negative control treatment group was 1.10%, and the increase in the average frequency of cells with micronuclei relative to the concurrent negative control group in the colchicine (positive control) treatment group (4.80%) was statistically significant (*p* ≤ 0.05, Figure 1A,D). No statistically significant increases in the percentage of cells with micronuclei in the test article-treated groups were observed at any concentration relative to the concurrent negative control group (*p* > 0.05, Figure 1A,D); the frequency of micronucleated cells was within the negative control data range reported in the literature (0.0–2.20%); and a concentration-related response was not observed. Based on these results, GlcNAc was concluded to be clearly negative for the induction of micronuclei in the test system of 24 h exposure in the absence of S9 metabolic activation.

### 3.3. GlcNAc Was Negative for the Induction of Micronuclei in the Polychromatic Erythrocytes of Mice

**Figure 2:**
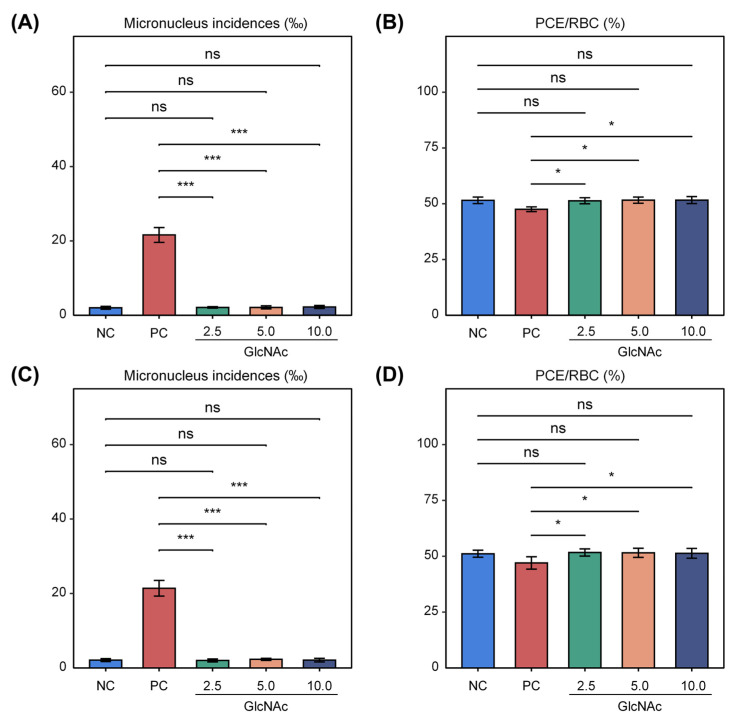
Effects of GlcNAc on micronucleus formation and PCE/RBC ratio in mice. (**A**) Micronucleus incidence in female mice. (**B**) PCE/RBC ratio in female mice. (**C**) Micronucleus incidence in male mice. (**D**) PCE/RBC ratio in male mice. Data are expressed as mean ± SD. Two-tailed Welch’s *t*-test was used to compare means between two independent groups. * *p* < 0.05; *** *p* < 0.001. NC: negative control; PC: positive control, treated with cyclophosphamide (40 mg/kg). PCEs: polychromatic erythrocytes; RBCs: total red blood cells. For each mouse, total of 10,000 PCEs were scored for micronuclei. ns, not significant (*p* > 0.05).

The effects of GlcNAc on micronucleus formation in mice are summarized in Figure 2. The incidences of micronuclei in female and male dose groups ranged from 2.0‰ to 2.3‰, with no significant differences compared with the negative control group. In contrast, the positive control groups showed markedly elevated micronucleus frequencies—21.6‰ in females and 21.4‰ in males—which were significantly higher than those in the negative control group (Figure 2A,C). Under the conditions of this assay, GlcNAc did not exhibit cytotoxicity, as the ratio of polychromatic erythrocytes (PCEs) to total red blood cells (RBCs) in both male and female groups remained above 20% of the values observed in the negative control group (Figure 2B,D). These findings indicate that GlcNAc did not induce a significant increase in micronucleus incidence in mouse polychromatic erythrocytes under the experimental conditions.

### 3.4. GlcNAc Did Not Affect Chromosomal Aberrations in Mouse Spermatocytes

The effect of GlcNAc on the chromosomal malformation rate in mouse spermatocytes is shown in Table 5. Compared with the negative control, there were no significant differences in the rate of chromosomally abnormal cells or in the early separation rates of autosomes and sex chromosomes among the GlcNAc dose groups. In contrast, the positive control showed significantly higher values than the negative control (*p* < 0.01). Moreover, all indices in every GlcNAc dose group were significantly lower than those in the positive control (*p* < 0.01). These results indicate that under the conditions of this test, GlcNAc did not have a significant impact on the occurrence of chromosomal aberrations in mouse spermatocytes.

## 4. Discussion

GlcNAc is an important amino sugar that is widely present in nature and is the basic constituent unit of biological macromolecules such as chitin. Currently, the edible safety, nutritional necessity, and application feasibility of GlcNAc have been widely recognized internationally, and the relevant production and application technologies are relatively mature. GlcNAc has been approved and marketed in multiple countries and regions worldwide, including China, Canada, and Korea, and is widely used in fields such as food, medicine, and cosmetics. Orally administered GlcNAc is hydrolyzed to glucosamine in both animals and humans [25].

Toxicological safety studies on glucosamine and GlcNAc have been carried out in publicly published reports. For example, glucosamine was not mutagenic in *E. coli* reverse mutation studies. A subchronic toxicity study of N-acetylglucosamine (GlcNAc) was conducted in groups of 10 male and 10 female F344 rats fed pelleted diets containing 0, 0.625, 1.25, 2.5, or 5% concentrations for 13 weeks, and it was concluded that orally administered GlcNAc exerts no obvious toxicity to F344 rats at concentrations up to 5% in the diet for 13 weeks [26]. The chronic toxicity and carcinogenicity of N-acetylglucosamine (GlcNAc) were examined in male and female F344 rats, and it was concluded that GlcNAc has neither toxic nor carcinogenic effects in F344 rats, with the no observed adverse effect levels (NOAELs) estimated from the chronic toxicity study being 5% in both sexes, equivalent to 2323 and 2545 mg/kg/day in males and females, respectively [27]. Although GlcNAc is regarded as non-toxic to humans, judging from the results of some toxicity studies, systematic safety evaluation for genotoxicity has been lacking. Thus, this study evaluated the potential genotoxicity of GlcNAc produced from the microbial fermentation of *Bacillus subtilis* BNZR 2-7 through a series of tests, including a bacterial reverse mutation assay (Ames test), in vitro micronucleus assay in Chinese hamster ovary cells, in vivo mammalian erythrocyte micronucleus test, and spermatocyte chromosome aberration test in mice.

GlcNAc was evaluated for the clastogenic/aneugenic potential in the in vitro micronucleus assay using Chinese hamster ovary (CHO-WBL) cells in the absence of activation systems for 3 and 24 h and in the presence of activation system for 3 h. The results indicated that the concurrent positive control articles, including cyclophosphamide monohydrate, mitomycin C, and colchicine, induced a significant increase in the percentage of cells with micronuclei, and the percentage of cells with micronuclei in the concurrent negative control was comparable to the range of negative control data reported in the literature, demonstrating the validity of the assay [28]. No significant increases in the percentage of cells with micronuclei were observed at any concentration in any of the three treatment series when compared to the concurrent negative control. The frequency of micronucleated cells was within the negative control data range reported in the literature, and a concentration-related response was not observed [28]. Based on the above data, GlcNAc was concluded to be negative for the induction of micronucleated cells in CHO-WBL cells under the conditions of the current study.

*Bacillus subtilis*, a GRAS-status bacterium demonstrated to be nonpathogenic and endotoxin-free, is widely used as a platform to produce industrially important biochemicals and pharmaceutical compounds [29,30,31,32]. Meanwhile, *Bacillus subtilis*, which is generally recognized as safe (GRAS), is a favorable industrial candidate as a cell factory because of its well-characterized genetics and metabolic robustness [33,34]. However, the construction of an *E. coli* triple-deletion mutant has been reported by several study groups, and the published data have shown that this strain has a severe growth defect when glucose is the sole carbon source [15,35,36]. In this study, we demonstrated the genotoxic safety of GlcNAc produced by *Bacillus subtilis* BNZR 2-7. Therefore, our study provided a fundamental basis for the application of GlcNAc produced by *Bacillus subtilis*.

While our findings support the genotoxic safety of the GlcNAc produced by *Bacillus subtilis* BNZR 2-7, several limitations should be noted. The substance evaluated in this study is GlcNAc, which is produced by microbial fermentation using *Bacillus subtilis* BNZR 2-7. The specific production method and fermentation strain of GlcNAc limit the generalizability of our findings because GlcNAc can be produced by multiple production methods and using various fermentation strains. Overall, the results of this study support the safety of GlcNAc produced by *Bacillus subtilis* BNZR 2-7 in food and can also serve as a reference for the safety of GlcNAc produced by other methods in food.

## 5. Conclusions

In summary, the GlcNAc produced by *Bacillus subtilis* BNZR 2-7 did not exhibit any mutagenic activity of bacterial species, did not induce micronucleated cells in CHO-WBL cells and the polychromatic erythrocytes of mice, and did not affect chromosomal aberrations in mouse spermatocytes. This product showed genotoxic safety in this comprehensive assessment in vitro and in vivo, and this study supports the safety of the GlcNAc produced by *Bacillus subtilis* BNZR 2-7 for inclusion in foods.

## Figures and Tables

**Table 1:** Positive control for tester strains in Ames test.

Tester Strains	S9 *	Positive Control	Dose Level
TA98, TA100, TA1535, TA1537	±	2-Aminoanthracene	2.0 μg/plate
WP2 uvrA	±	2-Aminoanthracene	20.0 μg/plate
TA98	−	2-Nitrofluorene	10.0 μg/plate
TA100, TA1535	−	Sodium Azide	1.0 μg/plate
TA1537	−	Acridine Mutagen ICR-191	1.0 μg/plate
WP2 uvrA	−	Methyl Methanesulfonate	2.5 μL/plate

* Tests were conducted under both conditions (±S9, a liver enzyme mix for metabolic activation), except where “−” indicates tests conducted under the condition without S9 only.

**Table 2:** The concentrations of GlcNAc for the in vitro micronucleus assay in Chinese hamster ovary cells.

Treatment Condition	Treatment Time	Harvest Time	Concentrations (μg/mL)
S9-activated	3 h	24 h	275, 550, 1100, 2212.1
Non-activated	3 h		275, 550, 1100, 2212.1
	24 h		275, 550, 1100, 2212.1

**Table 3:** Results of bacterial reverse mutation assay exposed to GlcNAc.

Groups		TA98	TA100	TA1535	TA1537	WP2 *uvr*A
**PC (+S9)**		620.0 ± 80.9	649.3 ± 167.4	81.3 ± 8.5	72.7 ± 21.1	120.0 ± 8.2
**NC (+S9)**		45.7 ± 5.7	119.7 ± 18.0	10.3 ± 3.5	10.7 ± 0.6	25.0 ± 3.5
**GlcNAc** **(+S9)** **μg/plate**	50	48.3 ± 5.0	114.0 ± 9.8	10.3 ± 1.5	10.3 ± 3.8	23.3 ± 2.5
	100	47.7 ± 4.7	125.7 ± 6.8	12.3 ± 0.6	8.0 ± 2.6	22.3 ± 3.2
	250	49.7 ± 5.7	117.3 ± 4.7	11.3 ± 2.9	9.7 ± 2.5	23.3 ± 4.2
	500	50.7 ± 3.8	121.0 ± 11.0	10.3 ± 3.5	11.7 ± 3.1	22.3 ± 2.9
	1000	48.7 ± 1.2	131.3 ± 4.7	11.0 ± 3.6	11.7 ± 3.1	21.7 ± 5.7
	2500	42.7 ± 1.5	130.0 ± 23.4	8.0 ± 1.7	9.0 ± 1.0	18.7 ± 1.5 *
	5000	45.7 ± 14.6	115.3 ± 13.3	10.7 ± 0.6	9.7 ± 1.2	20.0 ± 5.3
**PC (−S9)**		841.3 ± 50.0	485.3 ± 4.6	553.3 ± 148.4	233.3 ± 6.7	455.3 ± 103.9
**NC (−S9)**		35.0 ± 3.6	151.0 ± 7.0	12.7 ± 5.9	9.7 ± 2.9	18.3 ± 2.3
**GlcNAc** **(−S9)** **μg/plate**	50	38.0 ± 7.0	148.7 ± 22.3	10.7 ± 3.5	10.0 ± 3.5	18.0 ± 7.5
	100	37.0 ± 7.0	140.0 ± 16.5	15.0 ± 2.6	7.0 ± 0.0	18.7 ± 3.8
	250	32.7 ± 5.9	127.3 ± 9.1 *	10.7 ± 4.2	9.3 ± 0.6	23.0 ± 1.0
	500	34.3 ± 4.6	111.7 ± 10.7 *	11.3 ± 6.1	13.0 ± 1.7	22.7 ± 1.2
	1000	43.3 ± 5.0	111.0 ± 6.9 *	8.0 ± 1.0	8.3 ± 0.6	17.7 ± 3.1
	2500	31.7 ± 5.7	116.3 ± 9.7 *	11.7 ± 1.5	10.7 ± 2.1	19.0 ± 2.0
	5000	36.7 ± 11.0	108.0 ± 19.3 *	9.7 ± 5.1	9.3 ± 1.5	16.3 ± 4.2

* *p* < 0.05 compared to the negative control. NC: negative control; PC: positive control. All concentrations of GlcNAc and the NC demonstrated statistically significant differences (*p* < 0.001) compared to the PC for all tester strains.

**Table 4:** Result of in vitro micronucleus assay in Chinese hamster ovary cells exposed to GlcNAc.

Group	Dose Level (μg/mL)	Cytotoxicity (%)			Micronucleated Cells (%)		
		3 h + S9	3 h − S9	24 h − S9	3 h + S9	3 h − S9	24 h − S9
NC	0	0	0	0	1.00	0.95	1.10
PC	-	31	32	43	8.95	11.95	4.80
GlcNAc	550	0	2	3	1.10	0.80	0.75
	1100	2	−1	−1	1.40	0.55	0.80
	2212.1	3	−1	2	1.20	0.90	1.20

NC: negative control; PC: positive control, treated by cyclophosphamide monohydrate (2.5 μg/mL), mitomycin C (0.5 μg/mL), and colchicine (0.2 μg/mL); 3 h + S9: S9-activated 3 h treatment series; 3 h − S9: non-activated 3 h treatment series; 24 h − S9: non-activated 24 h treatment series.

**Table 5:** Results of spermatocyte chromosome aberration test in mice exposed to GlcNAc.

Group	Dose Level (g/kg)	Number of Cells Observed	Number of Sex Chromosome Monosomy	Number of Autosomal Monosomy	Number of Chromosome Aberration	Number of Chromosomal Abnormal Cells	Rate of Chromosomal Abnormal Cells (%)
NC	0.0	500	0	3	6	5	1.0
PC	-	500	0	13	65	53	10.6
GlcNAc	2.5	500	0	2	6	4	0.8
	5.0	500	0	2	7	5	1.0
	10.0	500	0	4	7	4	0.8

NC: negative control; PC: positive control, treated by cyclophosphamide (40 mg/kg).

## Data Availability

The original contributions presented in this study are included in the article. Further inquiries can be directed to the corresponding author.
